# Supplementary material for: The Interplay among Glucocorticoid Therapy, Platelet-Activating Factor and Endocannabinoid Release Influences the Inflammatory Response to COVID-19
Source: Viruses. 2023 Feb 19;15(2):573. doi: 10.3390/v15020573 (PMC9959303; doi:10.3390/v15020573)
Supplement: Supplementary file 1 [file viruses-15-00573-s001.zip › viruses-2165002-supplementary.pdf]

viruses MDPI

Supplementary Materials

Supplementary Table S1. Patient parameters according to clinical classification.

**Baseline variable**	**Healthy controls N= 35**	**COVID-19 All patients N= 200**	**Mild/Moderate N= 55**	**Severe/Critical N= 145**	***p Value***
**Demographic characteristics**					
Age, mean ± SD	37.7 ± 13.6	58.1 ± 18.7	42.7 ± 15.1	64.0 ± 16.6	[a, c, d]**<0.0001**; [b]>1.0
**Sex, n (%)**					
Man	18 (51.4)	108 (54.0)	25 (45.5)	83 (57.2)	[a]0.778; [b]0.580; [c]0.534; [d]0.135
Woman	17 (48.6)	92 (46.0)	30.0 (54.6)	62 (42.8)	
BMI (kg/m³)	25.7 ± 5.1	29.4 ± 7.1	27.5 ± 4.9	30.3 ± 7.7	[a]**0.016**; [b]0.685; [c]**0.003**; [d]0.272
**Comorbidities, n (%)**					
Hypertension	7 (20.0)	100 (50.0)	7 (12.7)	93 (64.1)	[a]**< 0.001**; [b]0.353; [c, d]**<0.0001**
Cardiovascular disorder	2 (5.7)	21 (10.1)	9 (16.4)	12 (8.3)	[a]0.379; [b]0,132; [c]0.611; [d]0.095
Diabetes *mellitus*	4 (11.4)	65 (32.5)	7 (12.7)	58 (40.0)	[a]0.011; [b]0.854; [c]0.001; [d]0.001
**Presenting symptoms, n (%)**					
Dyspnea	-	124 (62.0)	20 (36.4)	104 (71.7)	[d]**<0.0001**
Fever	-	67 (33.5)	6 (10.9)	61 (42.1)	[d]**<0.0001**
Myalgia	-	46 (23.0)	3 (5.5)	43 (29.7)	[d]**<0.0001**
**Laboratory findings, mean ± SD**					
Erythrocytes x 10^9/L	5.0 ± 0.5	4.2 ± 0.9	4.7 ± 0.6	4.0 ± 0.9	[a, c, d]**<0.0001**; [b]0.720
Hemoglobin (g/dL)	15.1 ± 1.3	12.5 ± 2.6	14.0 ± 1.9	11.9 ± 2.6	[a, c, d]**<0.0001**; [b]0.331
Leukocytes x 10^9/L	7.9 ± 2.2	1.1 ± 9.4	7.4 ± 2.1	12.4 ± 10.6	[a]**0.047**; [b]>1.0; [c]**0.0001**; [d]**<0.0001**
Neutrophils x 10^9/L	4.7 ± 1.8	8.6 ± 7.1	4.5 ± 1.9	10.1 ± 7.7	[a, c, d] **<0.0001**; [b] >1.0
Lymphocytes x 10^9/L	2.3 ± 0.6	1.5 ± 0.9	2.2 ± 0.8	1.2 ± 0.8	[a, c, d]**<0.0001**; [b]>1.0
Neutrophil/lymphocyte ratio	1.0 ± 0.9	1.5 ± 09	2.2 ± 0.8	1.2 ± 0.8	[a]**0.005**; [b, d]**<0.0001**; [c]0.629
Monocytes x 10^9/L	0.6 ± 0.2	0.5 ± 0.4	0.5 ± 0.2	0.5 ± 0.4	[a]0.199; [b]0.368; [c]0.243; [d]>1.0

Platelets x 10⁹/L	227.2 ± 45.2	255.3 ± 101.5	242.1 ± 72.0	260.4 ± 110.4	[a]0.866; [b, d]>1.0; [c]0.631
Glycemia (mg/dL)	88.1 ± 18.1	149.9 ± 82.4	109.2 ± 64.8	166.8 ± 83.2	[a, c, d]**<0.0001**; [b]0.414
PCR (mg/dL)	-	127.3 ± 102.6	84.7 ± 57.9	131.4 ± 105.2	[d]>1.0
Lactate (mg/dL)	-	14.5 ±19.2	5.7 ± 1.3	15.0 ± 19.6	[d]**0.011**
Ferritin (µg/L)	194.7 ± 143.1	252.1 ± 247.8	246.5 ± 236.8	567,2 ± 868.7	[a,b,c,d]>1.0
Oxygen Saturation	97.1 ± 3.7	90.1 ± 8.6	96.7 ± 3.4	87.7 ± 8.6	[a, c, d]**<0.0001**; [b]>1.0
PaO2/FiO2 ratio	-	153.6 ± 136.1	80.4 ± 160.7	181.3 ± 114.4	[d]**<0.0001**
**Hospital support, n (%)**					
Ward	-	89 (44.5)	12 (21.8)	77 (53.1)	[d]**<0.0001**
Intensive care unit (ICU)	-	68 (34.0)	-	68 (46.9)	-
**Hospitalization data, n**					
Days in Hospital	-	8.7 ± 8.1	2.6 ± 6.9	11.0 ± 7.3	[d]**<0.0001**
Days from symptom onset to recruitment	-	5.3 ± 3.2	3.4 ± 3.3	6.1 ± 2.8	[d]**<0.0001**
**Respiratory support received (%)**				-	
High flow nasal cannula	-	57 (28.5)	8 (14.6)	49 (33.8)	[d]**0.007**
Oxygen masks/ Noninvasive	-	35 (17.5)	2 (3.6)	33 (22.8)	[d]**0.001**
Invasive ventilation	-	61 (30.5)	-	61 (42.1)	-
**Medications, n (%)**					
Glucocorticoid	-	123 (61.5)	20 (36.3)	103 (71.0)	[a,b,c,d]**<0.0001**
Azithromycin	-	113 (56.5)	25 (45.5)	88 (60.7)	[d]0.052
Ceftriaxone	-	100 (50.0)	10 (18.2)	90 (62.1)	[d]**<0.0001**
Oseltamivir	-	69 (34.5)	10 (18.2)	59 (40.7)	[d]**0.002**
Colchicine	-	5 (2.5)	-	5 (3.5)	[d]0.163
CQ / HCQs	-	35 (17.5)	3 (5.5)	32 (22.1)	[d]**0.005**
Invermectin	-	7 (3.5)	6(10.91)	1 (0.7)	[d]**0.001**
**Diagnostic Test, n (%)**					
RT-PCR	35 (100)	190 (95.0)	45 (84.9)	145 (100)	-
Serological assays	-	10 (5.0)	10 (18.1)	-	-

Patient data were compared using the *Chi-square* test. or Fisher's exact test for categorical variables and one-way analysis of variance (ANOVA) Mann-Whitney. Nonparametric *t*-test was used for continuous variables. $p < 0.05$ was considered statistically significant. Abbreviations: Standard deviation (SD); percentage (%).[a]Comparisons between the Healthy controls versus COVID-19 all patients; [b]Healthy controls versus Mild/Moderate group; [c]Healthy controls versus Severe/Critical group; [d]Mild/Moderate versus Severe/Critical groups.

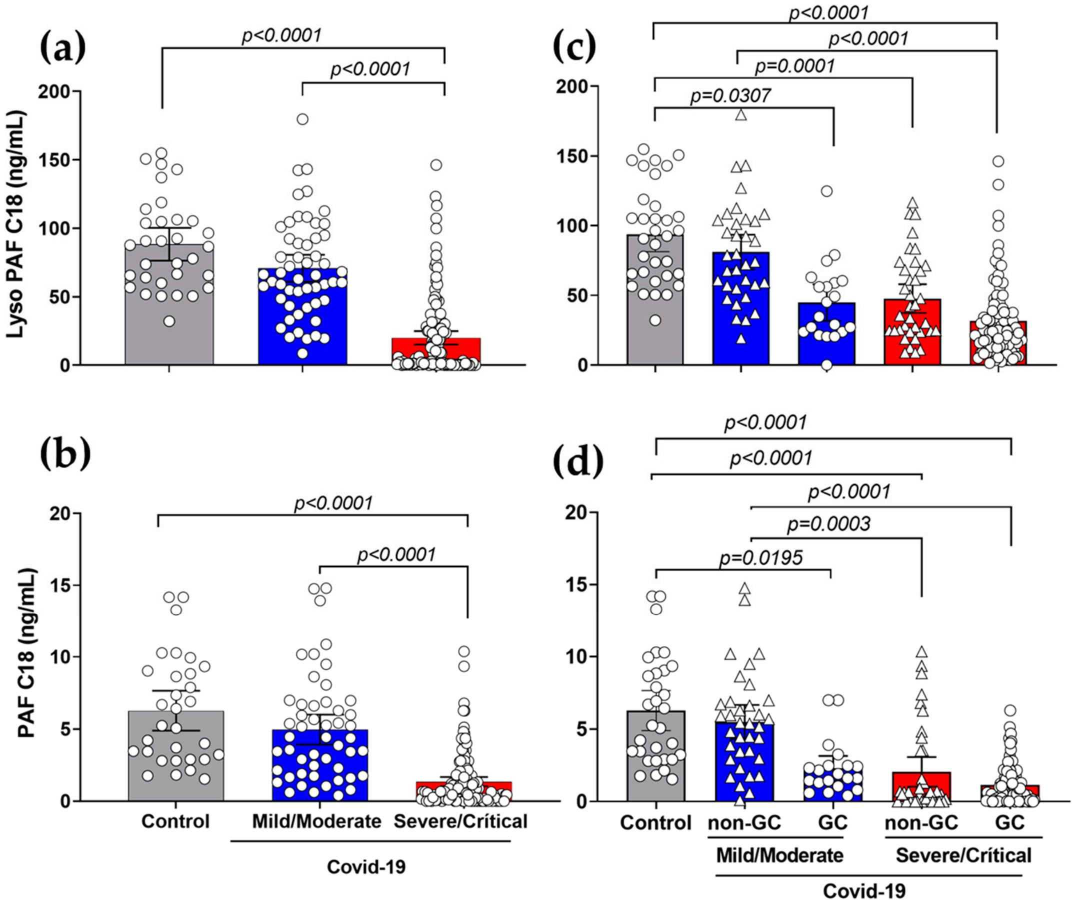

Figure S1. Production of Lyso-PAF C18 and PAF C18 in COVID-19 patients with the use of GCs**.** Levels of **(a)** Lyso-PAF (18:0) and **(b)** PAF (18:0) in COVID-19 patients (mild/moderate (*n*= 55) and severe/critical (*n*= 145) compared to healthy controls (*n*=35). COVID-19 patients who used or not GCs and segregated into mild/moderate (non-GC *n*= 35 vs GC *n*= 20) and severe/critical (non-GC *n*= 42 vs GC *n*= 103) show significant differences in **(c)** Lyso-PAF (18:0) and **(d)** PAF (18:0) compared to healthy controls. Statistical analyzes were performed using the Kruskal-Wallis multiple comparison test (non-parametric). followed by Dunn's post-test. Data are expressed as median in boxplot graphs with minimum and maximum values with a confidence interval of +/- 95%. Significance levels shown are based on statistically significant *p*-values between groups with *p*-value < 0.05. Area ratio: area ratio between the analyzer and the internal standard.

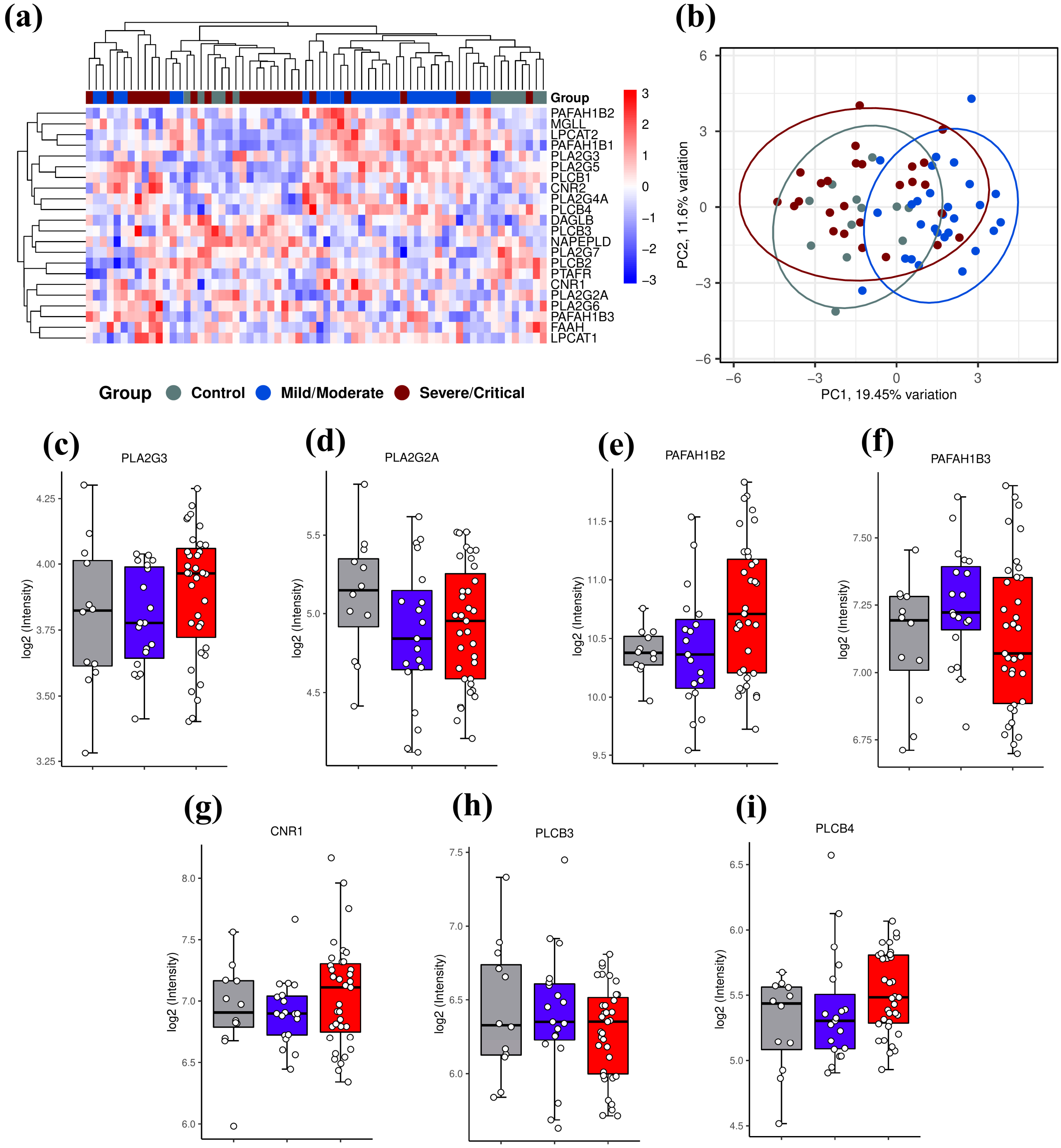

Figure S2. Gene expression of enzyme and receptor involved in the eCBs and PAF pathway in patients with COVID-19. **(a)** Unsupervised hierarchical heat map for gene expression of different patient groups according to disease severity (control (*n*=12); mild/moderate (*n*=26); and severe/critical (*n*=28)). Color gradient correspond to *Z-score* of log2 of normalized expression for each gene. **(b)** PCA plot and clustering of patients groups and control (95% confidence interval), including all genes displayed in the heat map, performed with PCAtools R library. Individual gene expression for: **(c)** PLA2G3; **(d)** PLA2G2A; **(e)** PAFAH1B2; **(f)** PAFAH1B3; **(g)** CNR1; **(h)** PLCB3; and **(i)** PLCB4, comparing the groups about the use of GC: Control (*n*=12), COVID-19 non-CG (*n*=19), and COVID-19 GC (*n*=35). The log2 of normalized gene expression profiles for analyzed groups are showed as boxplots. Table S2 contained gene fold change values, nominal and FDR adjusted *p*-values obtained from the differential expression analysis between the analyzed groups.

Supplementary Table S2. Differential gene expression results of enzyme and receptor involved in the eCBs and PAF pathways.

Gene Symbol	COVID-19 (non-GC) *vs* Control			COVID-19 (GC) *vs* Control			COVID-19 (GC) *vs* COVID-19 (non-GC)		
	FC	*p-value*	FDR	FC	*p*-value	FDR	FC	*p-value*	FDR
**CNR1**	-0.01	0.939	0.997	0.13	0.311	0.433	0.14	0.199	0.299
**CNR2**	0.26	0.064	0.723	0.36	0.005	0.020	0.10	0.357	0.471
**DAGLB**	0.17	0.195	0.850	0.06	0.619	0.719	-0.11	0.272	0.384
**FAAH**	-0.04	0.737	0.976	-0.09	0.362	0.486	-0.05	0.524	0.630
**LPCAT1**	0.20	0.172	0.841	-0.05	0.718	0.800	-0.25	0.030	0.067
**LPCAT2**	-0.29	0.209	0.858	0.83	0.001	0.001	1.12	< 0.001	< 0.001
**MGLL**	-0.08	0.829	0.985	0.89	0.011	0.035	0.97	0.004	0.005
**NAPEPLD**	-0.01	0.952	0.997	-0.53	0.004	0.017	-0.52	0.002	0.005
**PAFAH1B1**	-0.14	0.350	0.908	0.46	0.001	0.005	0.60	< 0.001	< 0.001
**PAFAH1B2**	0.03	0.892	0.991	0.37	0.035	0.083	0.34	0.021	0.051
**PAFAH1B3**	0.13	0.171	0.841	0.02	0.811	0.868	-0.11	0.136	0.221
**PLA2G2A**	-0.25	0.102	0.777	-0.19	0.165	0.271	0.06	0.622	0.715
**PLA2G3**	-0.02	0.857	0.988	0.09	0.286	0.408	0.11	0.139	0.225
**PLA2G4A**	0.27	0.077	0.727	0.43	0.002	0.010	0.16	0.159	0.249
**PLA2G5**	-0.03	0.815	0.985	0.60	< 0.001	0.001	0.64	< 0.001	< 0.001
**PLA2G6**	0.20	0.120	0.801	-0.08	0.518	0.634	-0.28	0.006	0.020
**PLA2G7**	-0.16	0.543	0.947	-0.77	0.002	0.009	-0.60	0.004	0.013
**PLCB1**	-0.16	0.535	0.945	0.54	0.021	0.057	0.69	0.001	0.003
**PLCB2**	-0.51	0.019	0.610	-0.73	0.001	0.002	-0.21	0.198	0.299
**PLCB3**	-0.03	0.812	0.985	-0.15	0.250	0.370	-0.11	0.296	0.410
**PLCB4**	0.11	0.399	0.921	0.24	0.048	0.106	0.13	0.209	0.312
**PTAFR**	-0.76	0.001	0.177	-0.30	0.120	0.214	0.46	0.006	0.019

For each comparison between groups is showed the fold change (FC) in log2 scale, the nominal *p*-value, and FDR adjusted *p*-value shown in Figure 3 and Figure S3.

Supplementary Table S3. Information about *r* and *p*-values of the correlation matrix shown in Figure 4a.

Comparison	Row	Column	Correlation *r*	*p*-value
**1**	PAF C16	2-AG	0.052	0.615
**2**	IL-10	2-AG	0.296	0.004
**3**	IL-6	2-AG	-0.241	0.040
**4**	Lymphocytes	2-AG	0.235	0.020
**5**	Neutrophils	2-AG	-0.213	0.038
**6**	sTREM-1	2-AG	-0.232	0.020
**7**	IL-10	PAF C16	-0.137	0.180
**8**	IL-6	PAF C16	0.003	0.981
**9**	Lymphocytes	PAF C16	0.322	0.001
**10**	Neutrophils	PAF C16	-0.101	0.336
**11**	sTREM-1	PAF C16	-0.061	0.544
**12**	IL-6	IL-10	-0.055	0.643
**13**	Lymphocytes	IL-10	0.281	0.004
**14**	Neutrophils	IL-10	-0.151	0.142
**15**	sTREM-1	IL-10	-0.175	0.070
**16**	Lymphocytes	IL-6	-0.026	0.824
**17**	Neutrophils	IL-6	-0.037	0.756
**18**	sTREM-1	IL-6	0.076	0.513
**19**	Neutrophils	Lymphocytes	-0.394	6.530
**20**	sTREM-1	Lymphocytes	-0.013	0.895
**21**	sTREM-1	Neutrophils	0.025	0.807

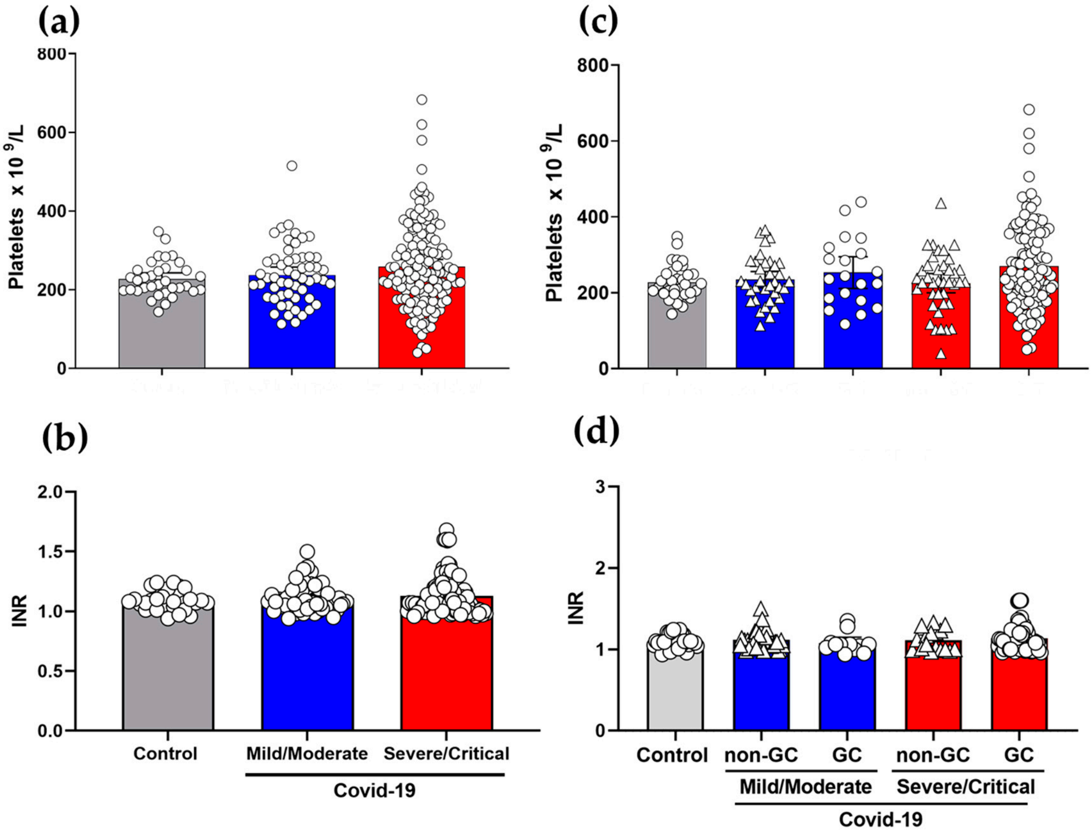

Figure S3. Thrombotic inflammatory markers in COVID-19 patients with GC treatment. The severity of COVID-19 did not influence the absolute values of (**a**) platelets and **(b)** INR (mild/moderate *n*=55 and severe/critical *n*=145) and the effect of GC treatment in the absolute values of (**c**) platelets and **(d)** INR with mild/moderate (non-GC *n*= 35 vs GC *n*= 20) and severe/critical (non-GC *n*= 42 vs GC *n*= 103) compared to healthy controls (*n* = 35). Statistical analyzes were performed using the Kruskal-Wallis multiple comparison test (non-parametric). followed by Dunn's post-test. Data are expressed as median in boxplot graphs with minimum and maximum values with a confidence interval of +/- 95%. Significance levels shown are based on statistically significant $p < 0.05$ between groups.

Supplementary Table S4. Information about *r* and *p*-values of the correlation matrix shown in Figure 5.

Comparison	Row	Column	Correlation	*p*-value
1	Lyso PC 20:4	Lyso PC 22:5	0.585	0.000
2	Lyso PC 18:2	Lyso PC 22:5	0.644	0.000
3	Lyso PC 16:0	Lyso PC 22:5	0.613	0.000
4	Lyso PC 18:0	Lyso PC 22:5	0.537	0.000
5	Lyso PE 22:5	Lyso PC 22:5	0.082	0.323
6	Lyso PE 18:1	Lyso PC 22:5	0.280	0.001
7	Lyso PE 20:4	Lyso PC 22:5	0.443	0.002
8	Lyso PE 16:0	Lyso PC 22:5	0.090	0.276
9	Lyso PE 18:0	Lyso PC 22:5	0.260	0.001

10	Lyso PE 18:2	Lyso PC 22:5	0.478	0.000
11	2-AG	Lyso PC 22:5	0.352	0.001
12	AEA	Lyso PC 22:5	-0.106	0.232
13	PAF C16	Lyso PC 22:5	0.431	0.000
14	Lyso PAF C16	Lyso PC 22:5	0.384	0.013
15	Lyso PAF C18	Lyso PC 22:5	0.313	2.224
16	PAF C18	Lyso PC 22:5	0.433	0.000
17	Lyso PC 18:2	Lyso PC 20:4	0.735	0.000
18	Lyso PC 16:0	Lyso PC 20:4	0.270	0.000
19	Lyso PC 18:0	Lyso PC 20:4	0.397	0.000
20	Lyso PE 22:5	Lyso PC 20:4	0.247	0.003
21	Lyso PE 18:1	Lyso PC 20:4	0.097	0.239
22	Lyso PE 20:4	Lyso PC 20:4	0.544	0.000
23	Lyso PE 16:0	Lyso PC 20:4	0.049	0.558
24	Lyso PE 18:0	Lyso PC 20:4	0.148	0.073
25	Lyso PE 18:2	Lyso PC 20:4	0.462	0.000
26	2-AG	Lyso PC 20:4	0.162	0.148
27	AEA	Lyso PC 20:4	0.007	0.935
28	PAF C16	Lyso PC 20:4	0.223	0.003
29	Lyso PAF C16	Lyso PC 20:4	0.479	0.000
30	Lyso PAF C18	Lyso PC 20:4	0.383	0.012
31	PAF C18	Lyso PC 20:4	0.416	0.002
32	Lyso PC 16:0	Lyso PC 18:2	0.560	0.000
33	Lyso PC 18:0	Lyso PC 18:2	0.548	0.000
34	Lyso PE 22:5	Lyso PC 18:2	0.123	0.138
35	Lyso PE 18:1	Lyso PC 18:2	0.278	0.001
36	Lyso PE 20:4	Lyso PC 18:2	0.387	0.116
37	Lyso PE 16:0	Lyso PC 18:2	0.158	0.055
38	Lyso PE 18:0	Lyso PC 18:2	0.186	0.024
39	Lyso PE 18:2	Lyso PC 18:2	0.470	0.000
40	2-AG	Lyso PC 18:2	0.225	0.043
41	AEA	Lyso PC 18:2	-0.116	0.189
42	PAF C16	Lyso PC 18:2	0.364	0.054
43	Lyso PAF C16	Lyso PC 18:2	0.394	0.005
44	Lyso PAF C18	Lyso PC 18:2	0.343	0.259
45	PAF C18	Lyso PC 18:2	0.323	1.623
46	Lyso PC 18:0	Lyso PC 16:0	0.655	0.000
47	Lyso PE 22:5	Lyso PC 16:0	-0.002	0.984
48	Lyso PE 18:1	Lyso PC 16:0	0.426	0.007
49	Lyso PE 20:4	Lyso PC 16:0	0.239	0.003
50	Lyso PE 16:0	Lyso PC 16:0	0.310	0.000

51	Lyso PE 18:0	Lyso PC 16:0	0.289	0.000
52	Lyso PE 18:2	Lyso PC 16:0	0.390	0.099
53	2-AG	Lyso PC 16:0	0.378	0.001
54	AEA	Lyso PC 16:0	-0.094	0.287
55	PAF C16	Lyso PC 16:0	0.510	0.000
56	Lyso PAF C16	Lyso PC 16:0	0.313	2.071
57	Lyso PAF C18	Lyso PC 16:0	0.281	0.000
58	PAF C18	Lyso PC 16:0	0.209	0.006
59	Lyso PE 22:5	Lyso PC 18:0	0.183	0.026
60	Lyso PE 18:1	Lyso PC 18:0	0.394	0.075
61	Lyso PE 20:4	Lyso PC 18:0	0.318	8.077
62	Lyso PE 16:0	Lyso PC 18:0	0.223	0.006
63	Lyso PE 18:0	Lyso PC 18:0	0.455	0.001
64	Lyso PE 18:2	Lyso PC 18:0	0.377	0.237
65	2-AG	Lyso PC 18:0	0.221	0.047
66	AEA	Lyso PC 18:0	-0.028	0.753
67	PAF C16	Lyso PC 18:0	0.369	0.038
68	Lyso PAF C16	Lyso PC 18:0	0.098	0.191
69	Lyso PAF C18	Lyso PC 18:0	0.029	0.699
70	PAF C18	Lyso PC 18:0	0.151	0.048
71	Lyso PE 18:1	Lyso PE 22:5	0.546	0.000
72	Lyso PE 20:4	Lyso PE 22:5	0.653	0.000
73	Lyso PE 16:0	Lyso PE 22:5	0.625	0.000
74	Lyso PE 18:0	Lyso PE 22:5	0.591	0.000
75	Lyso PE 18:2	Lyso PE 22:5	0.600	0.000
76	2-AG	Lyso PE 22:5	0.089	0.486
77	AEA	Lyso PE 22:5	-0.099	0.335
78	PAF C16	Lyso PE 22:5	-0.128	0.132
79	Lyso PAF C16	Lyso PE 22:5	-0.158	0.063
80	Lyso PAF C18	Lyso PE 22:5	-0.093	0.274
81	PAF C18	Lyso PE 22:5	0.007	0.939
82	Lyso PE 20:4	Lyso PE 18:1	0.559	0.000
83	Lyso PE 16:0	Lyso PE 18:1	0.815	0.000
84	Lyso PE 18:0	Lyso PE 18:1	0.818	0.000
85	Lyso PE 18:2	Lyso PE 18:1	0.663	0.000
86	2-AG	Lyso PE 18:1	0.047	0.711
87	AEA	Lyso PE 18:1	-0.238	0.020
88	PAF C16	Lyso PE 18:1	0.163	0.055
89	Lyso PAF C16	Lyso PE 18:1	-0.087	0.308
90	Lyso PAF C18	Lyso PE 18:1	-0.009	0.917
91	PAF C18	Lyso PE 18:1	0.084	0.332

92	Lyso PE 16:0	Lyso PE 20:4	0.561	0.000
93	Lyso PE 18:0	Lyso PE 20:4	0.663	0.000
94	Lyso PE 18:2	Lyso PE 20:4	0.856	0.000
95	2-AG	Lyso PE 20:4	0.132	0.297
96	AEA	Lyso PE 20:4	-0.069	0.503
97	PAF C16	Lyso PE 20:4	0.308	0.000
98	Lyso PAF C16	Lyso PE 20:4	0.350	2.299
99	Lyso PAF C18	Lyso PE 20:4	0.319	0.000
100	PAF C18	Lyso PE 20:4	0.433	0.015
101	Lyso PE 18:0	Lyso PE 16:0	0.787	0.000
102	Lyso PE 18:2	Lyso PE 16:0	0.656	0.000
103	2-AG	Lyso PE 16:0	-0.040	0.751
104	AEA	Lyso PE 16:0	-0.177	0.084
105	PAF C16	Lyso PE 16:0	0.123	0.147
106	Lyso PAF C16	Lyso PE 16:0	-0.052	0.543
107	Lyso PAF C18	Lyso PE 16:0	0.079	0.351
108	PAF C18	Lyso PE 16:0	0.024	0.787
109	Lyso PE 18:2	Lyso PE 18:0	0.690	0.000
110	2-AG	Lyso PE 18:0	-0.069	0.587
111	AEA	Lyso PE 18:0	-0.206	0.044
112	PAF C16	Lyso PE 18:0	0.190	0.024
113	Lyso PAF C16	Lyso PE 18:0	0.035	0.684
114	Lyso PAF C18	Lyso PE 18:0	0.074	0.387
115	PAF C18	Lyso PE 18:0	0.191	0.026
116	2-AG	Lyso PE 18:2	0.122	0.339
117	AEA	Lyso PE 18:2	-0.115	0.264
118	PAF C16	Lyso PE 18:2	0.360	1.275
119	Lyso PAF C16	Lyso PE 18:2	0.320	0.000
120	Lyso PAF C18	Lyso PE 18:2	0.344	3.129
121	PAF C18	Lyso PE 18:2	0.427	0.024
122	AEA	2-AG	0.876	-0.019
123	PAF C16	2-AG	0.003	0.335
124	Lyso PAF C16	2-AG	0.044	0.229
125	Lyso PAF C18	2-AG	0.300	0.119
126	PAF C18	2-AG	0.250	0.133
127	PAF C16	AEA	0.015	0.862
128	Lyso PAF C16	AEA	-0.122	0.170
129	Lyso PAF C18	AEA	-0.172	0.052
130	PAF C18	AEA	-0.021	0.822
131	Lyso PAF C16	PAF C16	0.624	0.000
132	Lyso PAF C18	PAF C16	0.552	0.000

133	PAF C18	PAF C16	0.550	0.000
134	Lyso PAF C18	Lyso PAF C16	0.743	0.000
135	PAF C18	Lyso PAF C16	0.635	0.000
136	PAF C18	Lyso PAF C18	0.471	0.000
107	Lyso PC 20:4	Lyso PC 22:5	0.585	0.000
108	Lyso PC 18:2	Lyso PC 22:5	0.644	0.000
109	Lyso PC 16:0	Lyso PC 22:5	0.613	0.000
110	Lyso PC 18:0	Lyso PC 22:5	0.537	0.000
111	Lyso PE 22:5	Lyso PC 22:5	0.082	0.323
112	Lyso PE 18:1	Lyso PC 22:5	0.280	0.001
113	Lyso PE 20:4	Lyso PC 22:5	0.443	0.002
114	Lyso PE 16:0	Lyso PC 22:5	0.090	0.276
115	Lyso PE 18:0	Lyso PC 22:5	0.260	0.001
116	Lyso PE 18:2	Lyso PC 22:5	0.478	0.000
117	2-AG	Lyso PC 22:5	0.352	0.001
118	AEA	Lyso PC 22:5	-0.106	0.232
119	PAF C16	Lyso PC 22:5	0.431	0.000
120	Lyso PAF C16	Lyso PC 22:5	0.384	0.013
121	Lyso PAF C18	Lyso PC 22:5	0.313	2.224
122	PAF C18	Lyso PC 22:5	0.433	0.000
123	Lyso PC 18:2	Lyso PC 20:4	0.735	0.000
124	Lyso PC 16:0	Lyso PC 20:4	0.270	0.000
125	Lyso PC 18:0	Lyso PC 20:4	0.397	0.000
126	Lyso PE 22:5	Lyso PC 20:4	0.247	0.003
127	Lyso PE 18:1	Lyso PC 20:4	0.097	0.239
128	Lyso PE 20:4	Lyso PC 20:4	0.544	0.000
129	Lyso PE 16:0	Lyso PC 20:4	0.049	0.558
130	Lyso PE 18:0	Lyso PC 20:4	0.148	0.073
131	Lyso PE 18:2	Lyso PC 20:4	0.462	0.000
132	2-AG	Lyso PC 20:4	0.162	0.148
